# An Integrated Framework to Achieve Interoperability in Person-Centric Health Management

**DOI:** 10.1155/2011/549282

**Published:** 2011-07-24

**Authors:** Fabio Vergari, Tullio Salmon Cinotti, Alfredo D'Elia, Luca Roffia, Guido Zamagni, Claudio Lamberti

**Affiliations:** DEIS-ARCES, Università di Bologna, 40126 Bologna, Italy

## Abstract

The need for high-quality out-of-hospital healthcare is a known socioeconomic problem. Exploiting ICT's evolution, ad-hoc telemedicine solutions have been proposed in the past. Integrating such ad-hoc solutions in order to cost-effectively support the entire healthcare cycle is still a research challenge. In order to handle the heterogeneity of relevant information and to overcome the fragmentation of out-of-hospital instrumentation in person-centric healthcare systems, a shared and open source interoperability component can be adopted, which is ontology driven and based on the semantic web data model. The feasibility and the advantages of the proposed approach are demonstrated by presenting the use case of real-time monitoring of patients' health and their environmental context.

## 1. Introduction

Worldwide healthcare standards are considered important indicators of human progress and civilization as they strongly affect both the economy of countries and the quality of life of citizens [1]. While citizens' expectations from healthcare systems increase, healthcare is facing a high risk of severe decay in the near future, as demographic changes are causing the rise of radical costs and staff shortages in the healthcare system of many countries. The population is ageing in many geographic areas and is growing in others; the percentage of 65+ population in the EU15 will grow from the current 17.1% to the 25% expected in 2030 and the life expectancy will go up to 81.4 years in 2030 from the 75.9 years of 2008. Concurrently, the number of people with chronic diseases, with comorbidities, or with some kind of impairment is increasing. Chronic diseases represent the greatest cause of early death and disability. Cardiovascular disease (CVD) is the leading cause of death in Europe and in the industrialized countries. Accordingly, healthcare and social costs are exploding from a worldwide average of  9% of the gross domestic product (GDP) in 2005 to 11% expected in 2015. Most EU member states spend around 30%–40% of the total health expenditure on the elderly population and long-term care (as an example, Europe spends around 3% of the GDP for treating CVD) [1]. 

The Institute for Healthcare Improvement reports that many healthcare systems around the world will become unsustainable by 2015 and the only way to avoid this critical situation is to implement radical changes. As a consequence, the healthcare system is subject to reform in many countries. The challenge is to improve care efficiency and effectiveness and to support sustainability of healthcare evolution. The goal is to manage increasing costs, and reduce unnecessary tests by having access to all relevant data and promoting integration of diagnosis and treatment. The common approach to improve the quality of the care process is to enable service access “at any time and in any place” and to move from “how to treat patients” to “how to keep people healthy and prevent illness.” Emphasis is put on prevention instead of treatment, supporting early discharge, less costly recovery, and rehabilitation at home [2].

Information and communications technology (ICT) plays an important role in this change. Particularly, ICT contributes to reduce the distance between the user and the clinician, by enabling telemonitoring, which is based on the integration of healthcare, telecommunications, and information technologies. Telemonitoring enables continuous observation of patients' health status and offers the opportunity to collect and analyse large amounts of data regarding the patients and their clinical history. Different solutions exist and they meet specific requirements concerning security, privacy, usability, robustness, safety, and data traceability. 

Already in the year 2000, state-of-the-art analysis of remote care solutions demonstrated the benefits of telemedicine to healthcare costs [3]. Still, there is a recognized barrier to healthcare radical innovation and associated cost-effectiveness improvement: this barrier is the fragmentation of healthcare solutions and the lack of interoperability at many levels [4]. Bringing interoperability into the healthcare system is a great challenge as it requires innovation not only in healthcare technology in general but also in management and working style [5].

This paper focuses on one technological aspect, which is information level interoperability, and claims that the wide adoption of interoperability platforms is the way to open innovation in healthcare. This will be demonstrated by showing how an interoperability platform developed within the framework of a European project [6] can enforce information interoperability between different healthcare “legacy” solutions.

 The paper is organized as follows.  Section 2 reviews the history and current research in out-of-hospital care and shows the impact of lack of interoperability in health care systems.  Section 3 describes the proposed approach and summarizes CHIRON's vision [7]. The proposed interoperability platform, developed within SOFIA project is then described in Section 4. In Section 5, we present a general scenario based on this platform to provide innovative services obtained by the concurrent monitoring of people's physiological parameters together with their surrounding environmental conditions. 

Section 6 describes our interoperable and cross-domain application, together with its architecture and its design process. In Section 7 the conclusions are drawn.

## 2. Motivations and Related Works

 The need for telemedicine was already felt back in the seventies [8], but telemedicine started to develop fast only in the nineties, when data communication services over the telephone lines became widely available (e.g. [9]). 

By that time the social role of telemedicine in healthcare started to be investigated by public institutions like EU (1990) and World Health Organization (WHO/OMS); (1997) and, consequently, a wide number of telemedicine systems were then realized, the main purpose being to reduce the amount of time a primary physician must spend with the patient and to allow at the same time a high level of care. 

At the beginning, these telemedicine systems were dedicated to a specific application (e.g. telecardiology, teleradiology, telespirometry, and teledialisis). Then, with the advent of internet-enabled platforms, including mobile platforms, and with the development of location-based services, remote collection of contextualized data from sensors (on-body, stationary, located at home, or in other environments) became possible. Sensors and also innovative actuators opened up new healthcare scenarios and new opportunities in e-health services, many of which are investigated by publicly cofunded research projects.

Classical telemedicine projects are focused on dedicated solutions. Their target is mostly limited to sensor data collection and delivery to medical personnel. Data mining and decision making are marginally addressed. These kinds of systems are usually based on specific sensors. It is difficult to add devices from different manufacturers, with different protocols or based on different technologies. For example, OLDES (Older people's e-services at home) [10] is an EU cofunded project under the IST Programme aiming to plan and develop a technological solution for teleassistance and teleaccompany; it supports a predefined set of specific instruments and VoIP technology. A similar project is AILISA [11], a French initiative promoting an experimental platform to evaluate monitoring technologies for elderly people in their homes. All of these projects benefit from the progress of ICT technologies; they are addressed to the elderly people and implement the classical telemedicine approach, and they are not able to exploit all the potential offered by such technologies because they do not inherently provide an automatic decision flow nor do they offer interoperable solutions.

These features can be found in “health smart homes” targeting elderly and impaired people at home. Smart homes are presented as a “dwelling incorporating a communications network that connects the key electrical appliances and services and allows them to be remotely controlled, monitored, or accessed” [12]. In particular a health smart home should ensure “an autonomous life at home to people who would normally be placed in institutions” [13]. These solutions are based on ambient intelligence research results and try to adapt the technology to people's needs by building on three basic concepts: ubiquitous computing, ubiquitous communication, and intelligent user interfaces. A proof of the importance of the integration of context data into remote health care applications is the growing number of projects and systems with this purpose. In this respect, we can cite MonAMI [14], Smart Medical Home [15], Aware Home [16], TAFETA [17], Gator Tech House [18], Duke Smart Home [19] and B-Live system [20]. Other solutions are discussed in [21]; this survey presents an international selection of advanced smart home solutions.

The common denominator within the above-mentioned solutions is that every system maintains its own proprietary protocols, platforms, information store, and data representation models. Therefore the information produced remains inside the original system implementation leading to an inherent fragmentation along the health care cycle.

This fragmentation, due to the lack of an ontology or a common standard, is the source of unnecessary overhead and it is also a barrier to healthcare service innovation. 

If health and context data are shared and made interoperable, then they might be combined and the new knowledge thus generated could support innovative applications for the benefit of multiple institutions and users. Interoperability is becoming a more and more relevant requirement for which solutions have been proposed, for example, by the database community [22] even before the start of the smart houses research era. Interoperability between heterogeneous and distributed systems handling information originated by the physical space has been increasingly investigated since the emergence of Weiser vision on pervasive computing [23]. The IEEE defines interoperability as “the ability of two or more systems or components to exchange information and to use the information that has been exchanged” [24].

With reference to the platforms for smart and physical space related applications (as also telemedicine applications), based on [25], the clear distinction between the following separated conceptual interoperability levels should be considered: communication level, service level, and information level interoperability.

Specifically, information (or semantic) interoperability is the shared understanding of information meaning. Service level interoperability is the ability for a system to share, discover and compose services. Communication level interoperability is interoperability at OSI levels 1, 2, 3, and 4 [26]. 

As already mentioned, this work focuses on information level interoperability and investigates the adoption in healthcare of an interoperability platform which is agnostic with respect to the service and communication levels used. The main benefit of the proposed solution is the possibility to easily create an open and dynamic smart environment where different actors and systems cooperate on the same information store to enrich the shared knowledge.

## 3. Scenario for Person-Centric Health Management 

The envisioned scenarios of high-quality and sustainable person-centric health management are clearly multidomain in nature, as they address the patient, the doctor, and the scientific community. Information level interoperability is an important concern since row data originates from heterogeneous devices that are inherently incompatible due to lack of standardization and because they are produced by a competing industry. 

Our vision is also shared by the partners of CHIRON, a 2010–12 EU project currently in progress. CHIRON's goal is to design an architecture solution for effective and person-centric health management along the complete health care cycle. The main challenge is to integrate the most recent patient information and their historical data into personal health systems and to transform collected information into valuable support for decision making [7]. To this end, several requirements should be met. First of all, data need to be gathered from multiple heterogeneous sources, (e.g. sensors, archives, and databases). Furthermore, in order to make them available in a meaningful and easy way, such data should be stored and uniformly represented in a data sharing platform. Once data are stored, some mechanisms to retrieve and analyze such data are needed. Finally, a shared metadata for information representation is required to harmonize the process of assessing the patient clinical situation and to support the doctor in his/her decision making process. 

In order to meet these requirements, CHIRON reference architecture defines the following three layers: the user plane, the medical plane, and the statistical plane.

(i) The user plane concerns interactions by and with the patients (monitoring and local feedback).
(ii) The medical plane concerns interactions by and with the doctors (assessment of clinical data, diagnosis, treatment planning and execution, and feedback to the patient).
(iii) The statistical plane concerns interactions with medical researchers (external knowledge management).

The integrated care cycle is based on the continuous interaction and exchange of information between the above-defined three planes. An innovative interoperability platform developed within the framework of SOFIA, another ARTEMIS JU project, may implement the core of CHIRON middleware layer. The proposed platform will contribute to the integrated care cycle, by managing an information space of portable and stationary sensors data, supporting different and heterogeneous measurement devices and actuators, and enabling personal profiling and a personalized approach in healthcare.

## 4. Information Interoperability and Interoperability Platform

This section describes the platform that ensures interoperability to the addressed health management scenario. 

The expected benefit is information integration to increase the knowledge about the patient and to facilitate the exchange of information between user, medical, and statistical planes.

The proposed platform consists of a set of tools supporting application development and application execution. It is open source [27] and—as a tool chain—it is called open innovation platform (OIP), while its runtime interoperability support is called Smart-M3 [28, 29]. Smart-M3 is based on the following concepts: information consumers and producers are decoupled and all the relevant information is stored on a shared information domain accessible by all the main actors. This approach opens an easy way to new unanticipated cross-domain applications as consumers do not need to know the details of the producer, but they only need to know how to access the shared information base which is based on an interoperable data model and shared semantics.

The OIP intends to introduce in this way a radical change to the traditional application scenario based on fixed business boundaries, as it inherently supports applications that can interoperate independently from their business/vendor/manufacturer origin.

The shared information domain accessible and understood by all authorized applications is called the smart space (SS). SS information is about entities existing in the physical environment, that is, the users, the objects surrounding them, the devices they use, or about the environment itself. Smart-M3 and the entire platform are developed with 15 principles in mind [30]. Out of these, the most relevant for our discussion are the *agnosticism*, the *notification*, the *evolvability*, the *extensibility,* and the *legacy *principles. The *agnosticism principle* states that the OIP should be agnostic with respect to use-cases, applications, ontology used to represent the information, programming languages, services and communication layers exposing the SS and hosting system. The *notification principle* ensures that clients may subscribe to be alerted upon specified events. The *evolvability principle* envisions that the OIP should provide means to support clients that adapt (i.e., “dynamically reconfigure” when changes in the SS information space occur). For example, if new relevant sensors are added to the SS, the application should benefit without the need to change the existing code. 

The *extensibility principle* calls for the inherent and efficient support to add features to the OIP, both at information and service level. Based on wise tradeoffs between the extensibility and the agnosticism principle, appropriate ontologies and data models could be defined, for example, to add access control and privacy policies and enforce authentication. Information security and privacy, as well as trust management, are fundamental qualities in a telemedicine scenario, that may become an OIP extension. The *legacy principle *states that existing (i.e., legacy) devices may exchange information with the SS with interface modules called “legacy adapters.” Smart space and legacy adapters and their architectural support in the OIP are conceptualized as follows.

**Figure 1:**
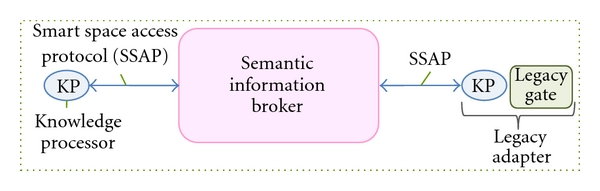
Smart M3 conceptual architecture the “smart space is in the SIB.

The OIP is built around its interoperable data sharing framework, named Smart-M3, and released by Nokia within SOFIA project. Smart-M3 defines two types of entities (Figure 1): the semantic information broker (SIB) and the knowledge processor (KP).

 The SIB is a digital entity where relevant real-world information is stored and kept up-to-date. It is an information store for sharing and governing all the data relevant for the domain of interest. The information model is a directed labeled graph corresponding to a set of RDF triples (resource description framework a basic semantic web standard [31]). The information semantics is specified by ontologies defined in OWL [31]. A query language [32] augmented by an inferential component provides reasoning support to applications. 

The KPs are software components interacting with the SIB and producing and/or consuming data. The legacy adapters are KPs that enable legacy SS-unaware devices to exchange information with the SIB (Figure 1). A KP exchanges data through a simple protocol named smart space access protocol (SSAP), an application layer protocol based on XML. The SSAP defines a simple set of messages (*join*, *insert*, *remove*, *update*, *query*, *subscribe*, *and leave*) that can be used over multiple connectivity mechanisms, such as TCP/IP, Bluetooth, OSGI, and NoTA [25]. The *join* is the first operation done by a KP in order to register itself to the smart space. After this operation, the KP can write or delete a sub-graph (i.e., a list of triple) using the *insert* or the *remove *primitives. The *update* is an atomic operation including an *insert* and a *remove* message. The KP can retrieve information from the SIB with the *query* operation. The SIB supports a number of different query languages including triple-based queries and Wilbur queries [32]. The *subscribe *operation allows a KP to specify conditions at information level that, when verified, are notified automatically according to the subscription notification paradigm. When a KP subscribes to a part of the graph, it receives a notification whenever such graph is modified; since the notification message is provided by the SIB with a short delay and contains details about the modifications that occurred, the KP is able to react promptly and in a specific way. The *leave* operation is used by a KP to specify the end of its session with the SIB. A KP that performs the *leave* has to join again in order to interact again with the SS. 

KPs may be developed using an application development kit (ADK), which is meant to increase developers' productivity by hiding the ontology and the SSAP protocol details, raising in this way the programming level of abstraction. Applications may be developed in several popular programming languages, including Python, ANSI C, C#, and Java.

## 5. Scenario Description

The addressed scenario consists of users living and moving in an environment monitored by environmental sensors. The user also relies on personal devices, among which are medical devices, both wearable and not wearable. The user moves freely inside this smart environment and all health and user's context data are continuously monitored and collected.

The first requirement to build a smart space out of this scenario is to create a shared and comprehensible digital description of all the entities that play a significant role in such an environment. In order to map each physical entity to its digital representation in the smart space, each physical entity needs to be uniquely identified. After that, each physical entity and its properties should be described with an appropriate data model. This description is created at smart space initialization time and concerns the users (both patients and doctors), their profiles, the devices, the environments, and all the relations between these main actors. Relations between entities may change dynamically and this may be reflected in the smart space manually using GUIs or automatically using some identification or localization technology. For example, in the proposed clinical scenario, the association of the same set of devices to different patients can be done explicitly with a dedicated GUI or it may be done automatically using the RFID technology as follows. The medical devices and an RFID reader are located on a desk. The patient registers himself just putting his RFID tag on the RFID reader; then all measures taken by the devices on the desk are associated to this patient until the RFID tag is removed. 

**Figure 2:**
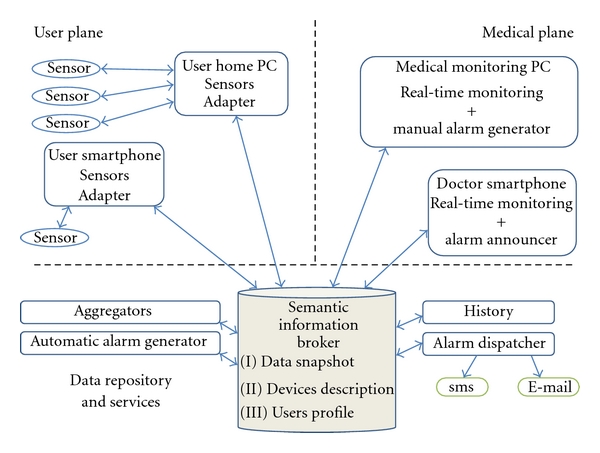
Linking medical and user plane.

The architecture of the proposed system is based on the platform described in Section 3 and it is shown in Figure 2. The OIP supports the user plane and its access from the medical plane. Different software agents (KPs) cooperate through the semantic information broker. 

In the user plane, data from heterogeneous sensors are collected by a PC or a smartphone and sent to the SIB. The sensors adapters are legacy adapter KPs (Figure 1) that could be seen as ontology driven translators: they enable the exchange of information between the SS and the legacy world.

The SIB is the core of the system; it stores and shares not only the data received from the devices but also all the information created during the initialization process. This implementation is consistent with the user profile concept proposed in CHIRON and called the “Alter Ego” [7]: the “Alter Ego” is an evolving virtual entity modeling all the relevant aspects related to the user health including medical history, habits, and functional health status. This entity has the capability of evolving and adapting over time to various domains and user conditions. Once generated, the profile is kept up-to-date automatically through the information provided by the multisensorial platform; in any case the doctor can add his inputs at any time. The user profile includes static/semistatic and dynamic parameters.

Based on the data collected by the sensors and on the knowledge of the relations between all the entities involved, new services can be devised. With reference to Figure 2, some examples of these services follow.

Aggregators are generic services consuming information from the SIB and enriching such information according to specific inference rules. Enriched information is stored back in the SIB in order to increase the knowledge and it includes, for example, indexes, aggregated parameters, or new semantic connections between existing entities. 

The automatic alarm generator is a service that generates an alarm condition on the patient profile as soon as at least one patient parameter goes out of the range prescribed by his/her profile. The conditions for raising an alarm are set by the doctors for every patient and are built into the patient profile. 

History service collects relevant data so that it can be statistically analyzed to discover or validate macroscopic relationships between patient profiles, diagnosis and the efficacy of the medical treatments. 

The alarm dispatcher service notifies an alarm status to the appropriate doctor using standard communication services like SMS and e-mail.

With reference to the medical plane, the following are examples of policies that could be implemented: medical staff could visualize all data collected from his/her patient through different platforms (PC or smartphone) in real time. Doctors could monitor their patients and specialists could be notified in case of alarm. Doctors could visualize, modify, and set a manual alarm according to a rule based-policy.

Starting from this scenario an application linking the user plane with the medical plane was implemented.

## 6. Application Design and Implementation

The implementation of a smart space application based on the proposed approach first requires an ontology to describe the domain of interest, then a set of KPs need to be developed in order to achieve the desired behavior.

**Figure 3:**
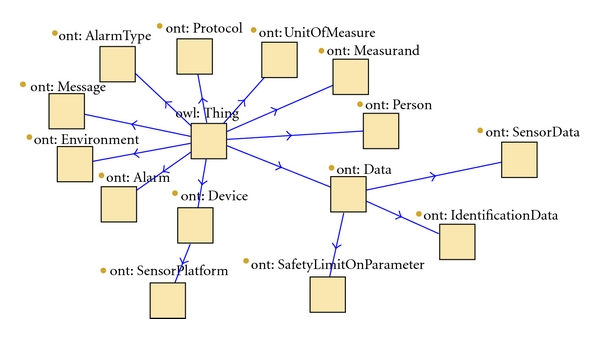
Ontology class tree.

 An ontology is a formal representation of the domain of interest made up of concepts and relations between them. When approaching ontology design, the designer must define classes, arrange them in a taxonomic (subclass-superclass) hierarchy, and define properties with their features (e.g., functional, inverse-functional, and symmetric). There is not a unique and optimized way to model a domain. On the contrary, there are always viable alternatives and the best solution depends on the application requirements and should be as intuitive, extensible, and maintainable as possible [33]. The ontology used in this application is an extension of an ontology modeled for a previous demonstration [34]. The ontology class tree is shown in Figure 3. The main classes are *Person*, *Environment, Data, Device,* and *Alarm*; they are all subclasses of *Thing*. The *Person* and *Environment* entities are self-explanatory. Devices are entities that can produce data or run KPs and are described by their characteristics, (e.g., MAC address, protocol). A *Data* entity is described by a value, a *Measurand*, a *Unit Of Measure,* and a timestamp. By modelling the data class in this way, we ensure that any KP that consumes sensor data would be able to take advantage of new sensor types without having to rethink the KP. Alarms are entities characterized by an *AlarmType*, for example, *HeartRateAlarm*. 

In our application, we have some users moving in an indoor space divided into two rooms and each environment is monitored by the sensors of a wireless sensor network (WSN); a ZigBee WSN, developed by Eurotech, is used to sense temperature, humidity, and the presence of water on the floor. In order to demonstrate the multivendor interoperability of the system, Intel iMote2 nodes were added to each room to further sense their environmental conditions. Both Eurotech WSN and iMote2 nodes send data to a local PC (i.e., the user's home PC) where there are two KPs, feeding the SIB, that is, one for the Eurotech WSN and one for the iMote2. The amount of sensors and the sensor network configuration do not need to be known a priori: in fact the system is able to benefit from certain sensors even if added at runtime. According to our ontology, when a sensor is added to the system, its KP inserts in the SIB a new instance of *Data* semantically related to the monitored entity; in this way, consumer KPs can discover all the data associated to a certain entity with just a query. 

 In our user case, we plan to do out-of-hospital monitoring of patients with a cardiovascular disease. The user is provided with the following medical devices: an A&D scale (UC-321PBT), an A&D sphygmomanometer (UA-767PBT), a Nonin finger pulse oximeter (Onyx II 9560), and a Zephyr BioHarness, which is a smart wearable device capable of collecting several vital signals.

All of these devices communicate via Bluetooth, each with its specific protocol. To satisfy the usability requirement and simplify user interaction, the user only needs to turn on the devices and take the measurements; data are sent to the repository without any further action.

 We achieve this functionality by exploiting the initial knowledge about the environment shared in the smart space. The only information needed, in fact, are the device's communication characteristics (MAC address and protocol) and a semantic connection associating the device to its current user. There is only one KP associated to each patient. This is the *PhysiologicalSensors* KP which communicates with all nearby Bluetooth devices semantically connected to the patient, and sends their data directly to the SIB. This KP works as follows: once launched, it queries the SIB for all information about the devices related to its user including their MAC addresses; then when one of these devices is discovered, it implements the proper protocol, parses the data, relates the data to its patient and updates the SIB. In the current implementation, this KP runs on a smartphone so that the sensors are wearable and the user can move while taking the measurements. In a different way, as shown in the previous section, a sensor can be stationary, shared, and only temporarily associated to a patient, for example, through the patient's RFID tag. While the RFID reader KP is responsible for handling the dynamic “semantic connections” between patient and instruments, the patient's *PhysiologicalSensors *KP dynamically searches all the associated sensors and gets their data as described. 

As user location needs to be known, each room is equipped with an RFID reader located next to the room entrance, while, as previously mentioned, each user has an own RFID tag for example attached to his/her smartphone. When a person enters a room, the corresponding tag is read and at information level the user is associated with the proper environment by the *Location KP.*

All information sent to the SIB can be used by multiple applications. To demonstrate the proposed person-centric health management approach, the following agents were implemented: the *ThomIndex*, the *AlarmGenerator*, the *AlarmNotifier*, the *HistoryLog*, the *HealthCareMonitoring* and the *ManualAlarmGenerator*. 

The *ThomIndex* KP evaluates the Thom Index (TI) which is a bioclimatic parameter measuring the perceived discomfort level originated by the combined effect of the environmental humidity and temperature [35]. The TI is evaluated as follows: the SIB is searched for all humidity and temperature data available; then the TI for each room is calculated based on the mean temperature and humidity values provided by the available and properly working sensors; eventually the TI associated to each room is stored back into the SIB. This KP is an example of “Aggregator KP” as it has the ability to aggregate raw data into higher level data with an increased semantic value.

**Figure 4:**
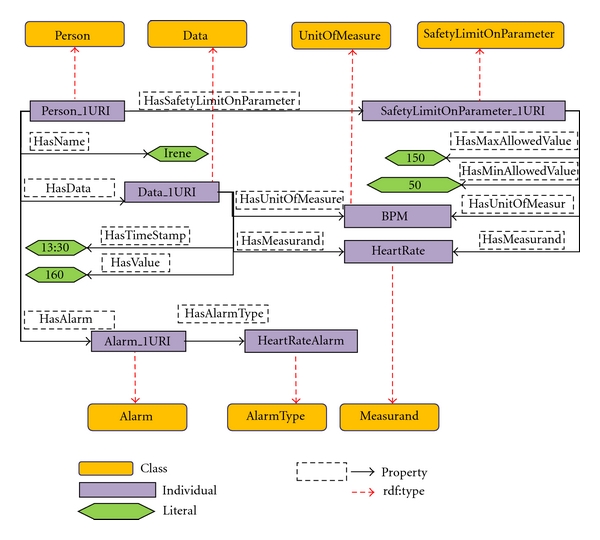
Example of a subgraph shared in the SIB.

The *AutomaticAlarmGenerator* KP is meant to publish the SIB alarm conditions detected according to properly specified policies. Currently, alarm conditions are very simple as they are just threshold based. If a user has a safety threshold inserted by the doctors and associated to certain parameters, then the KP performs appropriate subscriptions to the smart space in order to be notified of all updates of the sensible parameters. In Figure 4, a subset of the semantic graph used by the *AutomaticAlarmGenerator* KP is shown. Using the notation adopted in [36]: (i) the classes are represented in orange; (ii) each instance is connected to the classes it belongs to by the red dashed arrows representing the *rdf:type* property; (iii) classes, instances, and properties connecting them are uniquely identified by URIs (universal resource identifiers) whose semantic is commonly understood by all the software agents; (iv) literals (i.e., numerical and text values of properties noncorresponding to URIs) are shown in green. KPs access the graph by navigating through it with query operations and use the semantics of URIs to interpret the meaning of data. In Figure 4, the *Person* Irene has a safety threshold on her heart rate with maximum value 150 and minimum 50; as her data measuring heart rate exceeds the maximum value, an instance of alarm is generated and KPs subscribed to this information may find all the relevant data to react promptly to this situation. In our ontology, in fact, the instances of the *Person* class are also related to their respective doctor which can be contacted by another important KP: the *AlarmDispatcher*.

The *AlarmDispatcher* KP subscribes to the creation of *Alarm* instances, and, as soon as it is notified, it sends an email or an SMS with all related information to the appropriate doctor. Doctors' profiles include relevant personal information, particularly their email address and phone contact. Two alternative SMS sending solutions were implemented: the first runs on a Nokia N900 smartphone, while the second one on a PC connected to an embedded Siemens HC25 radio module. The *History* KP subscribes to all user data variations and logs the notified information in a file with an associated timestamp. 

The above KPs demonstrate our user plane implementation.

Moving to the medical plane, this is currently handled by a single KP named *HealthCareMonitoring *KP, which tracks all users' properties and allows the health care service to monitor a patient in realtime.

The KP allows the healthcare staff to select a patient, then creates a subscription to all of his/her relevant data. Therefore, it shows in real time all his/her data stored in the SIB, so not just physiological data, but also the person's environmental information as well, that is, health parameters together with the Thom Index and the environmental conditions of the place where she is located. 

**Figure 5:**
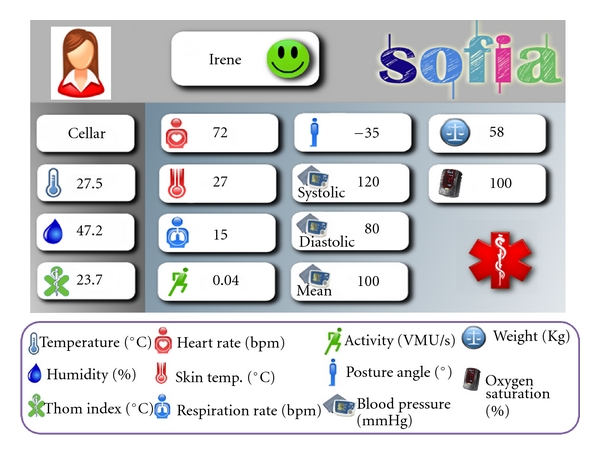
Health care monitoring GUI.

**Figure 6:**
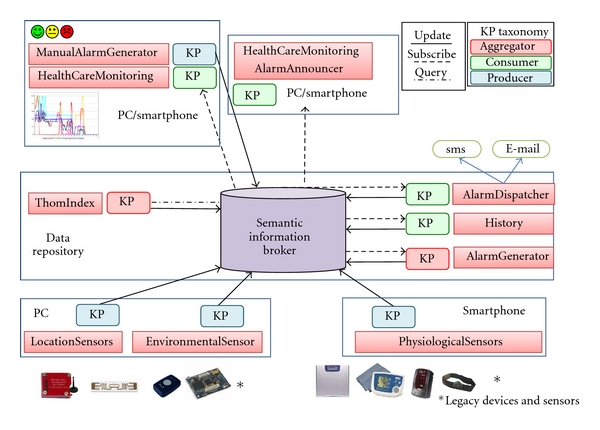
Interaction between KPs.

**Figure 7:**
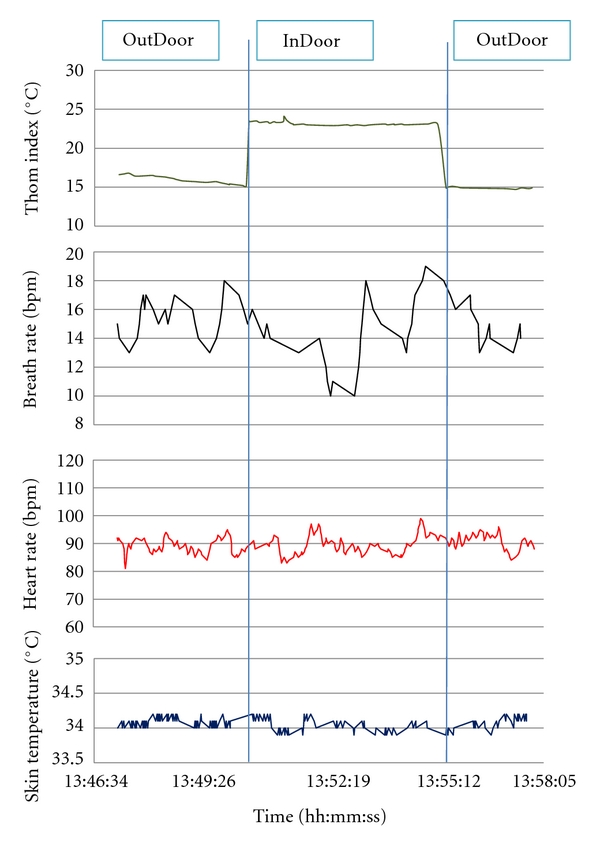
Health care monitoring session.

So far, based on the available sensors, the following health parameters may be collected: heart rate, respiration frequency, skin temperature, activity index, posture angle, weight, oxygen saturation, diastolic, systolic, and mean pressure. The *HealthCareMonitoring *KP detects alarm conditions and alerts the medical plane on top of the user plane *AutomaticAlarmGenerato*r KP.  Figure 5 shows the GUI of this KP. Furthermore, the healthcare staff, who monitors the patient's profile and current data, can also generate a manual alarm using the *ManualAlarmGenerator *KP. The interaction between the implemented KPs is depicted in Figure 6 while Figure 7 shows some preliminary data collected in our laboratory from a user wearing his BioHarness and walking around two rooms. The graph in Figure 7 shows the history of the following parameters over a short period of time during a hot summer day: environment Thom Index and patient's heart rate, respiration rate, and skin temperature.

## 7. Conclusions and Future Work

In this paper, a framework to support person-centric health management was presented and the proposed approach was demonstrated with a use case that focused on real-time monitoring of patients' health and environment.

In order to handle the heterogeneity of relevant information and to overcome the fragmentation of the instrumentation involved, a shared and open source interoperability component was proposed, which is ontology driven and based on the semantic web data model.

A significant set of multivendor physiological and environmental sensors was considered, and their data were collected, processed, and monitored.

With the proposed approach, information consumers and producers are decoupled and relevant information is stored on the shared information search domain offered by the interoperability platform and called “smart space.” The application functionalities are based on the cooperation of different software agents exchanging information through the smart space. Agents run on multivendor platforms based on different operating systems and written in various programming languages.

Smart space interoperability makes clear separation between device, service, and information level interoperability. The described work was focusing on information level interoperability, so primary requirements such as privacy and security were disregarded, being considered as service level qualities, therefore not relevant to the purpose of this paper. Consequently, the proposed solution now needs to be integrated within an SOA (i.e., a service-oriented architecture) and the plan is to integrate it within the architecture being developed within the already-mentioned CHIRON project. Here, standard ontologies will be adopted and the entire patient history together with the external information provided by the statistical plane will be offered in a unified view as a contribution to win the healthcare management challenge.
